# Telocinobufagin suppresses malignant metastasis of undifferentiated thyroid carcinoma via modulation of the *LARP1* ‐mTOR pathway

**DOI:** 10.1002/kjm2.12934

**Published:** 2025-01-09

**Authors:** Li‐Zhi Qiang, Shi‐Zhi Fang

**Affiliations:** ^1^ Department of Neck Surgery Sanming First Hospital Affiliated to Fujian Medical University Sanming China

**Keywords:** anaplastic thyroid cancer, *LARP1*, malignant metastasis, mTOR, Telocinobufagin

## Abstract

Metastasis is the trigger of death in anaplastic thyroid cancer (ATC) patients, yet the specific mechanisms at play are still largely enigmatic. While the involvement of *LARP1* in the metastatic process of various cancers has been documented, there is a noticeable gap in the literature regarding its potential influence on ATC metastasis. Molecular studies probed *LARP1* expression within ATC cells, with subsequent *in vitro* experiments examining the effects of *LARP1* on ATC cell metastasis and the mTOR signaling cascade. A suite of assays, including colony formation, scratch wound healing, transwell invasion, and cell adhesion, was used to assess cell growth, movement, invasion, and attachment. Western Blot determined the expression levels of epithelial–mesenchymal transition (EMT) markers (E‐cadherin, Vimentin, N‐cadherin) and proteins implicated in metastasis (MMP‐2, MMP‐9), along with mTOR and p‐mTOR. The affinity of Telocinobufagin (TBG) from Yuanhua Toad Essence for *LARP1* was investigated through molecular docking, with CETSA assays providing subsequent validation. Further cellular experiments substantiated the influence of TBG on ATC cell metastasis and modulation in the mTOR pathway. *LARP1* levels were heightened in ATC cells, and its depletion effectively curbs their proliferative, migratory, invasive, and adhesive activities. With *LARP1* knockdown, we also observed that the onset of EMT and metastatic processes was thwarted, as was the mTOR pathway. Subsequent research has uncovered that TBG formed a physical complex with *LARP1*, allowing it to target and suppress the mTOR pathway, thus preventing the metastasis of ATC. The simultaneous overexpression of *LARP1*, however, lessened the ability of TBG to inhibit ATC metastasis. This study highlights the importance of TBG binding to *LARP1* in the mediation of the mTOR signaling pathway, a key process in the inhibition of ATC cell metastasis. This discovery introduces a new target for the diagnosis of ATC and enlightens the consideration of TBG as a treatment for ATC metastasis.

## 1 INTRODUCTION

Thyroid carcinoma is among the most frequently occurring malignant endocrine tumors, with a rising trend in its incidence.^1^ Anaplastic thyroid cancer (ATC) is distinguished as the most malignant variant of thyroid cancer, with a swift progression, early propensity for invasion and metastasis, and a generally poor prognosis.^2^ The median survival span for ATC patients is alarmingly short, at 3–7 months.^3^ Despite the progress in ATC treatments over the years, the rapid disease progression and metastasis in the majority of patients have made it difficult to extend their survival, highlighting the lack of effective long‐term strategies^4^, ^5^, ^6^ There is an acute need to explore the molecular mechanisms behind aggressive metastasis of ATC to inform new directions in its management.

*LARP1*, or La‐related protein 1, is an RNA‐binding protein that has been conserved through evolutionary processes and is a component of the LARP superfamily.^7^ *LARP1* orchestrates the translation and stability of a specific class of mRNAs, known as terminal oligopyrimidine (TOP) mRNAs, which encode ribosomal proteins and translation factors and feature a 5′ TOP sequence.^8^ The correlation between *LARP1* and multiple human cancers is becoming more evident with each new research finding. Specifically, in colorectal cancer, *LARP1* has been confirmed as the RNA‐binding protein with the strongest upregulation, playing a role in fostering tumor expansion.^9^ Additionally, *LARP1* influences the cell cycle and metastasis in gastric cancer via the PI3K/AKT1 pathway.^10^ The specific expression profile and biological impact of *LARP1* in ATC are yet to be seen. Besides, the most frequently discussed regulatory mechanism for *LARP1* influencing TOP mRNA involves its phosphorylation by mTOR complex 1 (mTORC1).^11^ The function of *LARP1* as an integral mTORC1 signaling pathway substrate has been proven, and its modulation of mTOR mRNA transcript stability marks it as a primary agent in the translational governance of the mTOR pathway.^8^, ^12^ The mTOR signaling pathway is a definitive pathway in oncogenesis, critically involved in growth, metabolism, cell division, invasion, and malignant progression of human cancers.^13^, ^14^ Whether *LARP1* can mediate the mTOR signaling to control the malignant spread in ATC is not clear, which requires closer research.

More and more researchers are intrigued by cancer‐therapeutic potential of traditional Chinese medicine (TCM), which has seen extensive use in the oncology sector. Chan Su is a time‐honored anti‐cancer medicine that has proven its effectiveness against tumors.^15^ Telocinobufagin (TBG), one of the chemically active components extracted and refined from Chan Su, the secretions of the Bufo gargarizans, stands out for its diverse pharmacological actions, including cardiotonic and anti‐inflammatory effects.^16^, ^17^ Also, TBG displays impressive anti‐tumor activity. For example, in squamous cell carcinoma of the head and neck, it has been shown to suppress tumor growth and metastasis by decreasing the expression of PLK1.^18^ Its ability to obstruct the migration and invasion of breast cancer (BC) cells is also noted.^19^ However, the influence of TBG on ATC remains uncharted territory.

This research examined the influence exerted by *LARP1* on the metastatic behavior of ATC and the underlying molecular mechanisms, unveiling the interplay between *LARP1* and TBG. The data gleaned from our study suggest that *LARP1* serves as an oncogenic driver in ATC.

## 2 MATERIALS AND METHODS

### 2.1 Cell cultivation

The human thyroid epithelial cell line Nthy‐ori3‐1 (BNCC340487), along with the ATC cell lines KHM‐5M (BNCC100398) and CAL‐62 (BNCC359829), was procured from BeNa Culture Collection (BNCC, China). The ATC cell line 8505C (BFN60808779) was obtained from the Shanghai Cell Bank (China). Cultivation of Nthy‐ori3‐1, KHM‐5M, and 8505C was performed in RPMI‐1640 medium, while CAL‐62 was sustained in DMEM medium, both with a 10% fetal bovine serum (FBS) supplement. All the cells were cultivated in a humidified condition at 37°C with 5% CO_2_. ATC cells were treated with 0.5 μg/mL TBG for experimental operations 24 h later.^19^

### 2.2 Cell transfection

The shRNA targeting *LARP1* (sh‐*LARP1*), the overexpression construct for *LARP1* (oe‐*LARP1*), and their corresponding negative control vectors were all synthesized by GenePharma (China). These plasmids were then introduced into the ATC cells via the Lipofectamine 2000 reagent (Thermo Fisher Scientific, USA), and ATC cells with stably transfected *LARP1* were obtained. Post‐transfection, the cells were incubated for 48 h for subsequent assays.

### 2.3 CCK‐8 assay of cell viability

After being transfected for 48 h, the 8505C cells were seeded in a 96‐well plate at a density of 1 × 10^4^ cells per well. After being incubated in the CO_2_ incubator for 0, 24, 48, 72, and 96 h, the CCK‐8 reagent (10 μL per well) was added to the plate, and co‐incubated with the cells for 2 h. The OD values were measured at a wavelength of 450 nm.

The ATC cells (KHM‐5 M, CAL‐62, and 8505C) were seeded in a 96‐well plate at a density of 1 × 10^4^ cells per well. After being incubated for 48 h in the CO_2_ incubator, the cells were treated with different concentrations of TBG (0, 0.05, 0.1, 0.5, 1, 2 μg/mL) for 48 h and were further incubated for 48 h in a constant temperature incubator at 37°C. The CCK‐8 reagent (10 μL per well) was added to the plate, co‐incubated with the cells for 2 h, and the OD values were measured at a wavelength of 450 nm. Finally, the IC_50_ values in each group of cells were calculated using GraphPad Prism 8.0 (USA). Each group underwent three independent replicate experiments.

### 2.4 Quantitative reverse transcription polymerase chain reaction (qRT‐PCR)

The 2 × 10^6^ ATC cells collected from different treatment groups were subjected to lysis for total protein extraction using the RIPA lysis buffer (Beyotime, China). RNA concentration and purity were ascertained by a NanoDrop ND‐1000 spectrophotometer (Thermo Fisher Scientific, USA). The RNA was then reversed transcribed into cDNA through the HiFiScript All‐in‐one RT Master Mix for qPCR from Cwbio (China). The ensuing qRT‐PCR analysis was performed with the AceQ qPCR SYBR Green Master Mix from Vazyme (China) on the Applied Biosystems 7500 Fast Real‐Time PCR System (Thermo Fisher Scientific). The amplification cycle was set as follows: denaturation at 95°C for 30 s for one cycle, followed by annealing/extension at 95°C for 5 s and 60°C for 10 s, repeated for 40 cycles, employing *GAPDH* as our reference gene, and applying the 2^−ΔΔCT^ method for relative quantification. Table 1 lists the necessary primers. Three independent replicate experiments were conducted for each group.

**TABLE 1:** qPCR primers.

Gene	Sequence (5′ → 3′)	
*LARP1*	Forward primer	GCAACCTAAAGACACTAC
	Reverse primer	CCTCTTCTTCACTTCAATC
*GAPDH*	Forward primer	GGAAGCTTGTCATCAATG
	Reverse primer	CCCCACTTGATTTTGGAG

### 2.5 Western blot (WB)

The amassed ATC cells were lysed with RIPA buffer (Beyotime) to extract total proteins, which were then quantified using a BCA assay kit (Beyotime). Proteins were resolved on a 10% sodium dodecyl sulfate‐polyacrylamide gel electrophoresis (SDS‐PAGE) and transferred to polyvinylidene fluoride membranes (Millipore, USA), blocked with 5% milk at room temperature for 60 min, and incubated with primary antibodies against *LARP1* (ab317575; Abcam, UK), E‐cadherin (ab314063; Abcam), Vimentin (ab16700; Abcam), N‐cadherin (ab76011; Abcam), MMP‐2 (ab92536; Abcam), MMP‐9 (ab76003; Abcam), mTOR (ab134903; Abcam), p‐mTOR (#2971; Cell Signaling Technology, USA), and GAPDH (ab181602; Abcam) overnight at 4°C. After incubation with a secondary antibody, goat anti‐rabbit IgG (horseradish peroxidase) (ab6721; Abcam), for 1 h at room temperature, bands were visualized using a high‐sensitivity enhanced luminescence detection reagent kit (Beyotime) on a ChemiScope 6000 imaging system (Clinx, China). The experiments were independently repeated three times for each group.

### 2.6 Colony formation assay

ATC cells were seeded into 12‐well plates (400 cells per well) and cultured at 37°C in a 5% CO_2_ incubator for 14 days. Routine replacement of the culture medium was ensured. The colonies were fixed with 4% paraformaldehyde for 30 min, stained with 0.1% crystal violet for 30 min, and then counted under a camera. Three separate experiments were carried out for each group.

### 2.7 Wound healing assay

ATC cells were inoculated into a six‐well cell culture plate, each well receiving an allocation of 5 × 10^5^ cells. Cultivation ensued until the cells achieved a confluence level of 80%, at which point the medium was replaced with one devoid of serum. A manual incision was introduced by the methodical scraping of a 200 μL pipette tip, traversing the well in a straight trajectory, thereby disrupting the confluent cell monolayer. The cellular debris dislodged into the medium was subsequently flushed away with a phosphate‐buffered saline (PBS) rinse. Optical microscopy (Olympus, Japan) was employed to capture images at 0 and 24 h after the initiation of the scratch. The rate of wound closure was quantified by comparing the width of the scratch at both time points, applying the formula: wound healing rate (%) = ((wound width at 0 h − wound width at 24 h)/wound width at 0 h) × 100%. Three independent repetitions of the experiment were performed for each group.

### 2.8 Cell invasion detected by Transwell assay

For the assessment of cell invasiveness, we utilized Transwell chambers precoated with Matrigel (BD Biosciences, USA). ATC cells were harvested and resuspended in a serum‐free medium, followed by the inoculation of 2 × 10^5^ cells into the upper compartment of the Transwell. The lower compartment was filled with medium containing 10% FBS. After 24 h of incubation, cells that had invaded to the underside of the membrane were fixed with 4% paraformaldehyde for 5 min, rinsed with PBS three times, and stained with 0.1% crystal violet for 5 min. Finally, the stained cells were counted and their images recorded under an optical microscope (Olympus). Each group was subjected to three independent experimental repetitions.

### 2.9 Cell adhesion assay

ATC cells were seeded into a 96‐well plate that had been pre‐coated with fibronectin from BD Biosciences and supplemented with 1% FBS, at a seeding density of 3 × 10^4^ cells per well. After a 1‐h incubation period at 37°C, the plate was washed with PBS, fixed with a 4% paraformaldehyde solution, and stained with hematoxylin. The stained cells were then visualized and enumerated using an optical microscope (Olympus, Japan). Three independent experimental replicates were performed for each group.

### 2.10 Molecular docking simulation

To validate the binding affinity between a target protein and its active components, we performed molecular docking. The PDB files of the proteins were downloaded from RCSB, any ligands and non‐proteins were removed, and hydrogenation was applied. The SDF files of the small molecules were obtained from PubChem (https://pubchem.ncbi.nlm.nih.gov/) and converted to MOL2 format using Open Babel. Protein–drug interactions were then simulated on SwissDock (http://www.swissdock.ch/docking).

### 2.11 Cell thermal shift assay (CETSA)

Cells were subjected to a 24‐h treatment with TBG at a concentration of 0.5 μg/mL or an equivalent volume of dimethyl sulfoxide (DMSO), following which they were harvested and resuspended in PBS supplemented with a cocktail of protease inhibitors. This suspension was distributed equally across six PCR tubes and exposed to a heat shock at specific temperatures (37, 45, 47, 49, 51, and 53°C) for a duration of 3 min to effect protein denaturation. After that, the cells were lysed in NP‐40 lysis buffer and subjected to two rapid freeze–thaw cycles in liquid nitrogen. The lysate was then centrifuged (12,000 rpm, 4°C, 20 min), the supernatant was extracted, and an equivalent volume of 2 × SDS loading buffer was added. The protein content was subsequently determined by WB. Each group was tested in three independent experimental replicates.

### 2.12 Drug affinity responsive target stability (DARTS) assay

Based on the experimental method of the previous researchers,^20^ we employed DARTS to identify the interaction between TBG and *LARP1*. Cells were treated with TBG (0.5 μg/mL) or DMSO for 24 h. After treatment, cells were collected and lysed in a lysis buffer containing protease and phosphatase inhibitors. The collected lysates were divided into six groups. Each group of lysates was treated with different concentrations of pronase (0%, 0.01%, 0.03%, 0.1%, 0.3%, 1%) (Roche Diagnostics, Indianapolis, USA) for 30 min. After stopping the digestion by adding buffer, the samples were loaded onto SDS‐PAGE and stained with Coomassie Brilliant Blue for visualization. Three independent experiments were conducted for each group.

### 2.13 Data processing

Data from at least three experiments are shown as mean ± SD. GraphPad Prism 8.0 was used for statistical analysis, with t‐tests for two‐group comparisons and one‐way analysis of variance (ANOVA) for multiple groups. The significance is determined at *p* < 0.05.

## 3 RESULTS

### 3.1 *LARP1* promotes ATC migration and activates the mTOR signaling pathway

**FIGURE 1:**
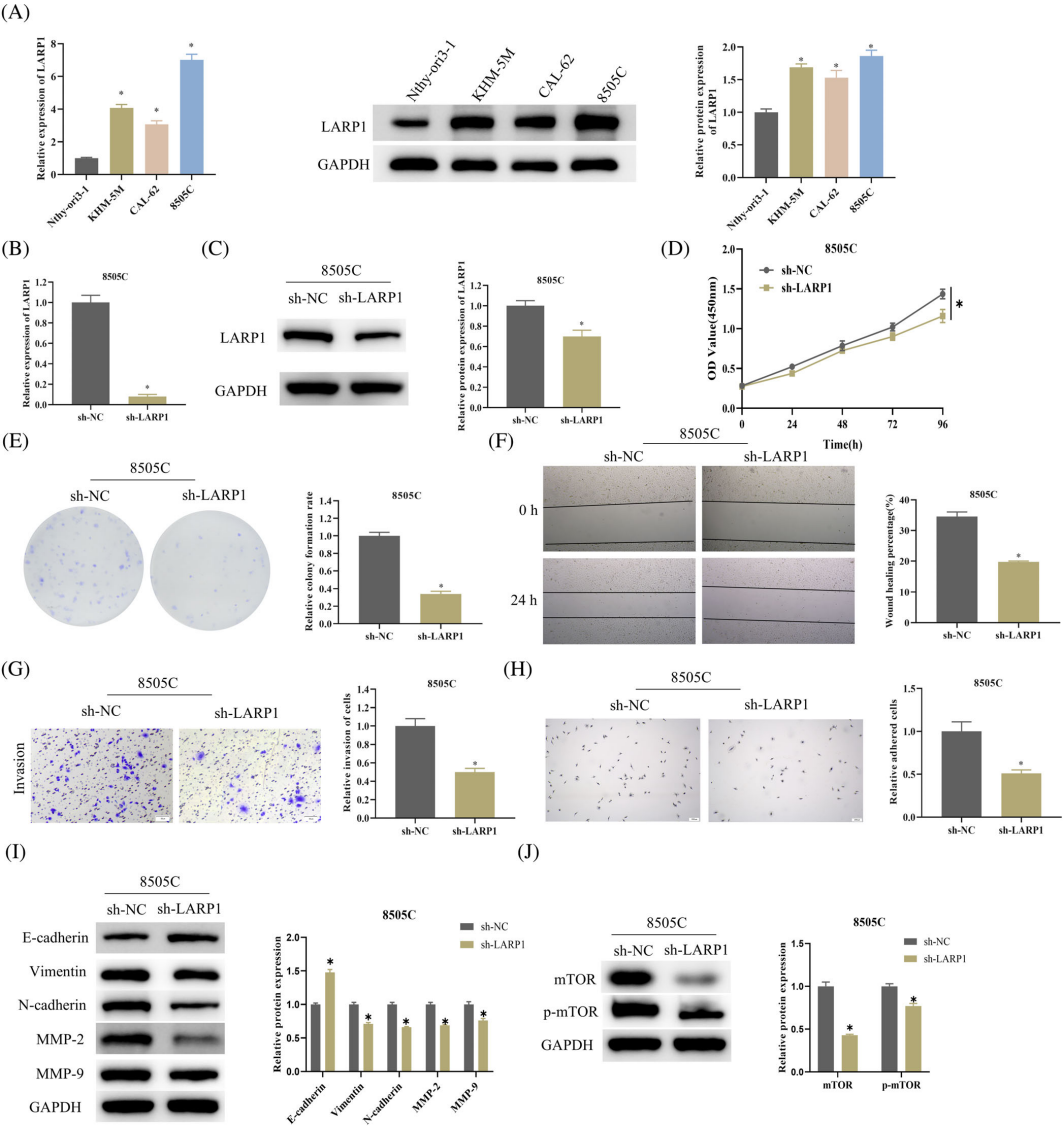
Effects of *LARP1* silencing on ATC metastasis and the mTOR signaling pathway. (A) qRT‐PCR and WB detection of *LARP1* mRNA and protein expression in normal human thyroid cells Nthy‐ori3‐1 and ATC cells KHM‐5M, CAL‐62, and 8505C. (B, C) Construction of sh‐NC and sh‐*LARP1* 8505C cell groups, with transfection efficiency assessed by qRT‐PCR and WB. (D) CCK‐8 assay of cell proliferation ability of sh‐NC and sh‐*LARP1* 8505C cell groups. (E) Colony formation assay for the proliferation of sh‐NC and sh‐*LARP1* 8505C cells, using crystal violet for staining. (F, G) Scratch healing and Transwell assays for the evaluation of sh‐NC and sh‐*LARP1* 8505C cell migration and invasion, respectively, using crystal violet for staining. (H) Cell adhesion assay for adhesion capability of sh‐NC and sh‐*LARP1* 8505C cell groups, using hematoxylin for staining. (I) WB analysis of epithelial–mesenchymal transition (EMT)‐related proteins (E‐cadherin, Vimentin, and N‐cadherin) and metastasis‐related proteins (MMP‐2 and MMP‐9) in 8505C cells. (J) WB detection of mTOR and p‐mTOR protein expression in 8505C cells. *n* = 3 independent replicate experiments, *p < 0.05. ATC, anaplastic thyroid cancer; WB, Western blot.

Using qRT‐PCR and WB, we compared *LARP1* levels in the normal thyroid cell line Nthy‐ori3‐1 against those in ATC cell lines KHM‐5M, CAL‐62, and 8505C, revealing elevated *LARP1* mRNA and protein expression in ATC cells relative to the normal cell lines (Figure 1A). This revelation sets the stage for an in‐depth exploration of *LARP1*'s role in the progression of ATC and utilizes shRNA to silence *LARP1* within the 8505C cell line, resulting in the formation of sh‐NC and sh‐*LARP1* groups. qPCR and WB confirmed successful knockdown by showing decreased *LARP1* mRNA and protein levels (Figure 1B,C). CCK‐8 experiments showed that the knockdown of *LARP1* significantly reduced the proliferative capacity of 8505C cells (Figure 1D). We next analyzed cell proliferation using a colony formation assay, showing *LARP1* knockdown seriously cut down the cloning efficiency of 8505C cells (Figure 1E). Scratch and Transwell assays indicated that *LARP1* reduction impeded both migration and invasion of 8505C cells (Figure 1F,G). Adhesion assays confirmed a notable drop in the count of attached 8505C cells with *LARP1* knockdown (Figure 1H). WB detected changes in epithelial–mesenchymal transition (EMT)‐associated proteins (E‐cadherin, Vimentin, and N‐cadherin) and metastasis‐associated proteins (MMP‐2 and MMP‐9), showing an accretion in E‐cadherin and a decrement in Vimentin, N‐cadherin, MMP‐2, and MMP‐9 upon *LARP1* knockdown (Figure 1I). Moreover, *LARP1* has been found to facilitate the onset and metastasis of various cancers via the mTOR pathway.^12^, ^21^ We suspected that *LARP1* could also control ATC progression through the agency of the mTOR pathway. We thus conducted WB to measure the expression of mTOR and its phosphorylated form, p‐mTOR, in distinct groups of 8505C cells, revealing a decrease in the expression of both proteins upon *LARP1* knockdown (Figure 1J). These findings corroborated the dual function of *LARP1* in the facilitation of ATC metastasis and the invigoration of the mTOR signaling pathway.

### 3.2 *LARP1* promotes the migration of ATC cells via activating the mTOR signaling pathway

**FIGURE 2:**
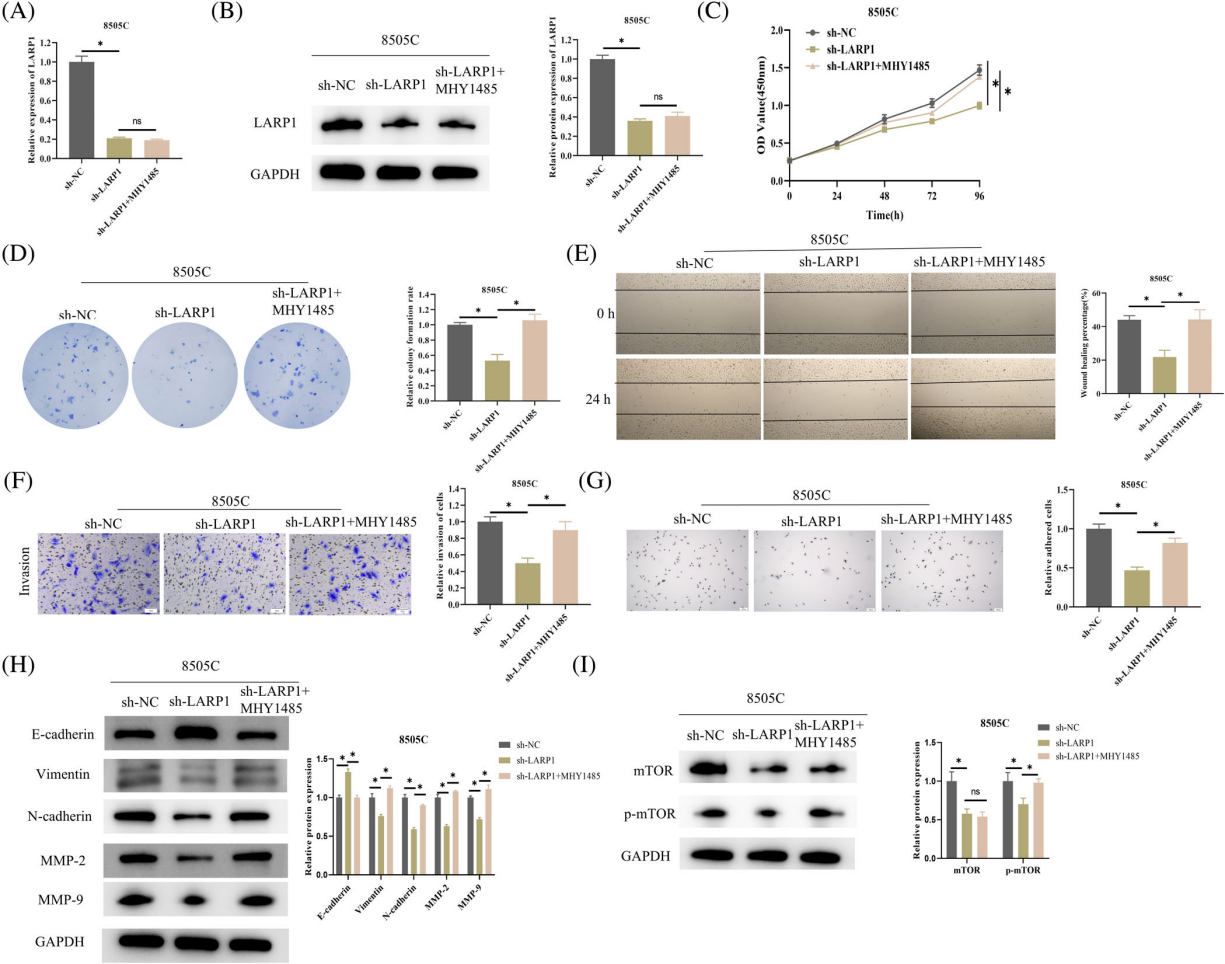
*LARP1* silencing inhibits ATC cell metastasis via the mTOR pathway. (A, B) Construction of sh‐NC, sh‐*LARP1* and sh‐*LARP1* + MHY1485 8505C cell groups, with transfection efficiency assessed by qRT‐PCR and WB. (C) CCK‐8 assay of cell proliferation ability of sh‐NC, sh‐*LARP1* and sh‐*LARP1* + MHY1485 8505C cell groups. (D) Colony formation assay for the proliferation of sh‐NC, sh‐*LARP1* and sh‐*LARP1* + MHY1485 8505C cells, using crystal violet for staining. (E, F) Scratch healing and Transwell assays for the evaluation of sh‐NC, sh‐*LARP1* and sh‐*LARP1* + MHY1485 8505C cell migration and invasion, respectively, using crystal violet for staining. (G) Cell adhesion assay for adhesion capability, using hematoxylin for staining. (H) WB analysis of epithelial–mesenchymal transition (EMT)‐related proteins (E‐cadherin, Vimentin, and N‐cadherin) and metastasis‐related proteins (MMP‐2 and MMP‐9) in 8505C cells. (I) WB detection of mTOR and p‐mTOR protein expression in 8505C cells. *n* = 3 independent replicate experiments, *p < 0.05. ATC, anaplastic thyroid cancer; WB, Western blot.

Existing literature indicates that the mTOR complex can regulate the cell cytoskeleton by altering the phosphorylation status of PKC and directly phosphorylating and activating Akt, thereby controlling EMT.^22^ To thoroughly confirm that *LARP1* regulates EMT markers and MMP‐2/MMP‐9 expression through the mTOR pathway, we designed the following experimental groups based on 8505C cells: sh‐NC, sh‐*LARP1*, sh‐*LARP1* + MHY1485 (mTOR pathway activator MHY1485, 10 μM). The expression of *LARP1* in different groups of 8505C cells was examined using qPCR and WB. The results revealed that knocking down *LARP1* significantly decreased both *LARP1* mRNA and protein expression in the cells, with no change upon MHY1485 treatment, indicating successful transfection (Figure 2A,B). CCK‐8 assay results demonstrated that knocking down *LARP1* significantly decreased the viability of 8505C cells, while further treatment with MHY1485 restored cell viability to control levels (Figure 2C). Subsequently, we assessed the proliferative capacity of the cells through a colony formation assay. Results showed that knocking down *LARP1* markedly reduced the proliferation of 8505C cells, whereas proliferation recovered to control levels after MHY1485 treatment (Figure 2D). Migration and invasion capabilities of 8505C cells were evaluated using scratch healing and Transwell assays, respectively. It was observed that knocking down *LARP1* inhibited the migration and invasion abilities of ATC cells, which were restored after treatment with MHY1485 (Figure 2E,F). Cell adhesion experiments indicated that knocking down *LARP1* reduced cell adhesion capacity, with a recovery to control levels after further treatment with MHY1485 (Figure 2G). WB analysis of EMT‐related proteins (E‐cadherin, Vimentin, and N‐cadherin) and metastasis‐related proteins (MMP‐2 and MMP‐9) in 8505C cells revealed that knocking down *LARP1* led to a significant increase in E‐cadherin protein expression and a decrease in Vimentin, N‐cadherin, MMP‐2, and MMP‐9 expression levels. Furthermore, treatment with MHY1485 restored the levels of these proteins to those of the control group (Figure 2H). WB experiments also indicated that knocking down *LARP1* reduced the protein expression of mTOR and p‐mTOR in 8505C cells, with p‐mTOR protein expression recovering to control levels after further treatment with MHY1485 (Figure 2I).

Additionally, based on 8505C cells, we designed the following groups: oe‐NC, oe‐*LARP1*, oe‐*LARP1* + Rapamycin (mTOR pathway inhibitor, 10 nM). Supplementary experiments confirmed that overexpression of *LARP1* significantly increased the viability, proliferation, migration, invasion, and adhesion capabilities of 8505C cells, promoting their EMT process. Furthermore, treatment with Rapamycin inhibited the enhancing effects of *LARP1* overexpression on the aforementioned cellular functions (Figure S1A–I). These results collectively suggested that *LARP1* could promote the migration of ATC cells through the mTOR signaling pathway.

### 3.3 TBG targets and binds to *LARP1*

**FIGURE 3:**
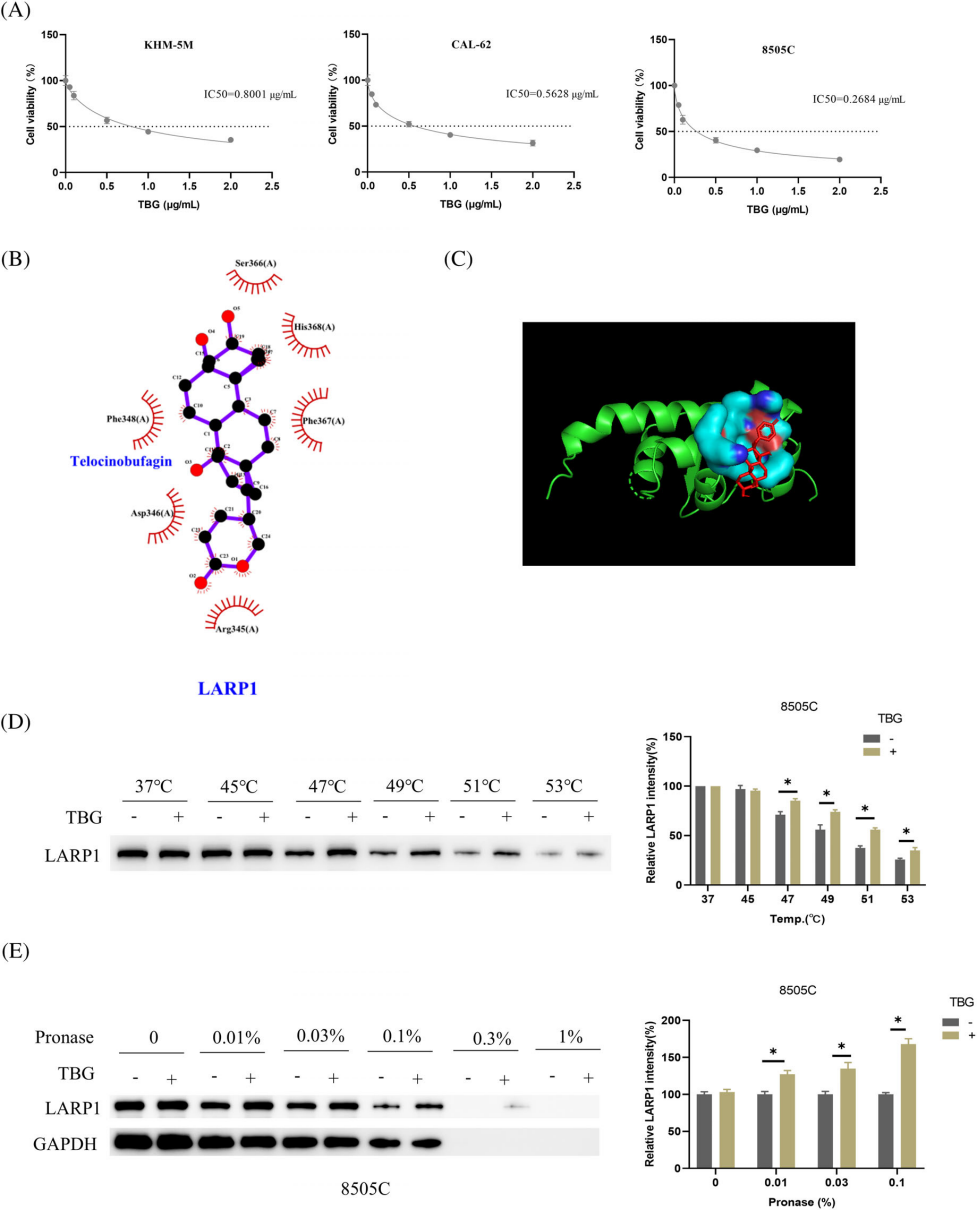
Physical interaction between TBG and *LARP1*. (A) CCK‐8 detection of the effect of TBG on ATC cells (KHM‐5M, CAL‐62 and 8505C) viability; (B, C) 2D and 3D diagrams of molecular docking simulation between TBG and *LARP1*. (D) Cellular thermal shift assay (CETSA) of ATC cell line 8505C exposed to dimethyl sulfoxide (DMSO) or 0.5 μg/mL TBG. (E) Drug affinity responsive target stability (DARTS) assay of ATC cell line 8505C exposed to DMSO or 0.5 μg/mL TBG. *n* = 3 independent replicate experiments, *p < 0.05. ATC, anaplastic thyroid cancer; TBG, Telocinobufagin.

It has been reported that TBG can inhibit the migration of BC cells via the mTOR pathway.^19^ Therefore, we investigated the impact of TBG on the growth of ATC cells through CCK‐8 experiments. The analysis results indicated that as the concentration of TBG (0, 0.05, 0.1, 0.5, 1, 2 μg/mL) increased, the vitality of ATC cells gradually decreased (Figure 3A). By considering the IC_50_ values of the three types of cells, we established the concentration of TBG for subsequent cellular functional experiments as 0.5 μg/mL. Meanwhile, *LARP1* is recognized for its role in facilitating the metastasis of non‐small cell lung cancer via the activation of the mTOR pathway.^12^ Thus, we postulated a connection between TBG and *LARP1*. To test this hypothesis, we performed molecular docking to simulate their interaction. The 2D representation is depicted in Figure 3B, with the corresponding 3D structure in Figure 3C, both revealing physical interaction. Further cellular thermal shift assays confirmed that TBG augments the thermal stability of *LARP1* at temperatures of 49, 51, and 53°C (Figure 3D), implying a direct binding interaction. The results of the DARTS assay showed that in the presence of TBG, the *LARP1* protein structural conformation was more stable and less susceptible to hydrolysis by broad‐spectrum protein hydrolases (Figure 3E). Our results substantiated the existence of a binding relationship between TBG and *LARP1*.

### 3.4 TBG inhibits ATC metastasis and blocks the mTOR signaling pathway

**FIGURE 4:**
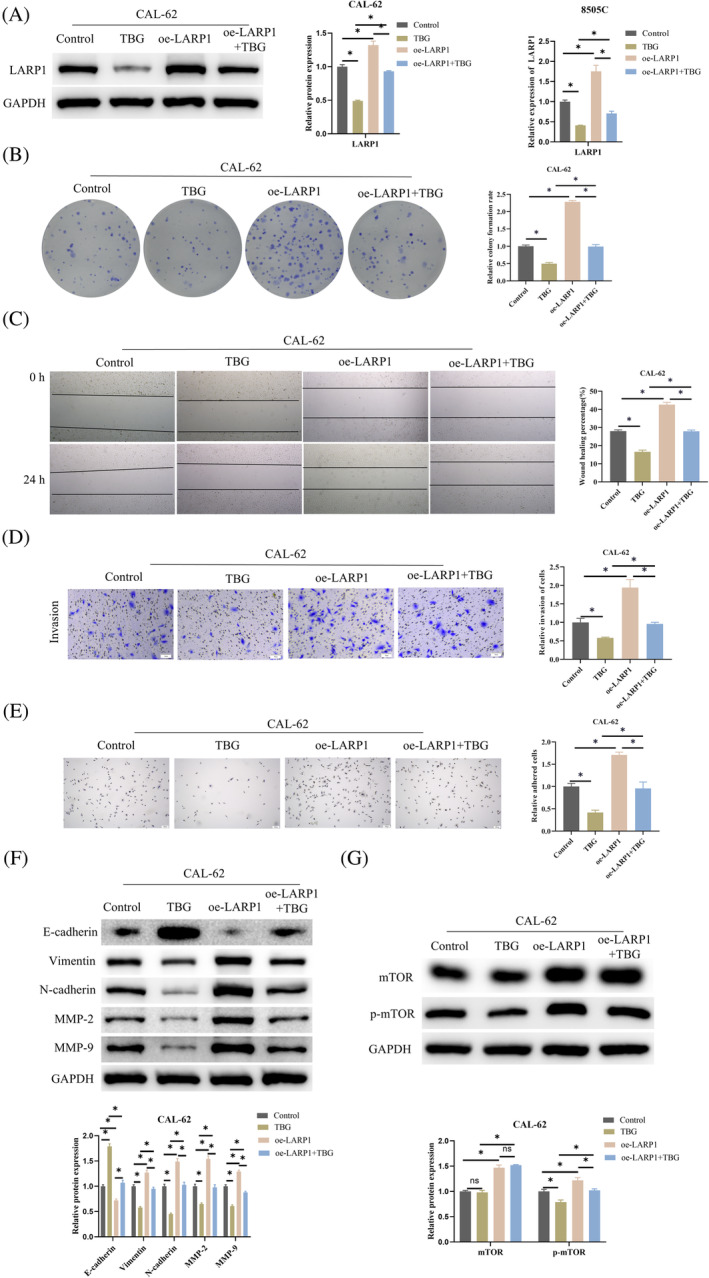
Effects of TBG on ATC metastasis and the mTOR signaling pathway in oe‐*LARP1*‐transfected cells. (A) The CAL‐62 cell groups, namely Control, TBG, oe‐*LARP1*, and oe‐*LARP1* + TBG, were subjected to qRT‐PCR and WB analysis to ascertain *LARP1* mRNA and protein expression. (B) Colony formation assay for CAL‐62 cell proliferation in Control, TBG, oe‐*LARP1*, and oe‐*LARP1* + TBG groups, using crystal violet for staining. (C, D) Scratch and Transwell assays for assessing migration and invasion of CAL‐62 cells in Control, TBG, oe‐*LARP1*, and oe‐*LARP1* + TBG groups, using crystal violet for staining. (E) Adhesion test for CAL‐62 cell adhesion in Control, TBG, oe‐*LARP1*, and oe‐*LARP1* + TBG groups, using hematoxylin for staining. (F) WB for epithelial–mesenchymal transition (EMT)‐related proteins (E‐cadherin, Vimentin, and N‐cadherin) and metastasis‐related proteins (MMP‐2 and MMP‐9) levels in CAL‐62 cells. (G) mTOR and p‐mTOR protein levels in CAL‐62 cells by WB. *n* = 3 independent replicate experiments, *p < 0.05. ATC, anaplastic thyroid cancer; TBG, Telocinobufagin; WB, Western blot.

To explore the impact of TBG on ATC metastasis, we established the following groups in CAL‐62 cells: Control, TBG, oe‐*LARP1*, and oe‐*LARP1* + TBG. Expression levels of *LARP1* across these groups were determined via WB and qRT‐PCR analyses, demonstrating a marked decrease in *LARP1* expression upon TBG treatment, with overexpression efforts rescuing *LARP1* to near‐normal levels (Figure 4A). A colony formation assay then appraised cellular proliferative abilities, indicating that TBG suppressed CAL‐62 cell proliferation, an effect that was attenuated by the overexpression of *LARP1* (Figure 4B). Scratch healing and Transwell assays revealed that CAL‐62 cell migration and invasion were greatly hindered by TBG treatment. However, the concurrent overexpression of *LARP1* alongside TBG mitigated this effect, returning migration and invasion to levels observed in the control group (Figure 4C,D). Moreover, TBG treatment diminished the adhesive capacity of CAL‐62 cells, an effect that was counteracted by the simultaneous overexpression of *LARP1*, which enhanced cell adhesion to a degree comparable to the control (Figure 4E). Employing WB, we quantified the levels of proteins associated with epithelial–mesenchymal transition (EMT) (E‐cadherin, Vimentin, and N‐cadherin) and metastasis (MMP‐2 and MMP‐9) in CAL‐62 cells. The introduction of TBG resulted in an upregulation of E‐cadherin and a downregulation of other proteins, with *LARP1* overexpression attenuating the impact of TBG on their expression (Figure 4F). Additionally, TBG treatment led to a reduction in phosphorylated mTOR expression without impacting total mTOR levels. Overexpression of *LARP1* in conjunction with TBG not only restored p‐mTOR levels but also enhanced mTOR expression (Figure 4G). Collectively, these results indicated that TBG could inhibit ATC metastasis and interrupt the mTOR signaling pathway.

## 4 DISCUSSION

ATC, a rare and progressively metastatic tumor, is associated with an extremely unfavorable prognosis.^23^ The obscure etiologies that catalyze the malignant progression of ATC underscore a quest to elucidate the mechanisms underpinning its malignant behaviors and to discover novel therapeutic targets. Our research has shed light on a new regulatory mechanism influencing the metastatic phenotype of ATC. TBG, an active component derived from Chan Su, modulates the mTOR signaling pathway by interacting with *LARP1*, thereby hindering the metastatic spread of ATC.

*LARP1* has been recognized as a driver of cancer progression, exhibiting increased expression in hepatoblastoma,^24^ lung cancer,^25^ and multiple myeloma,^26^ among other cancers. Our study also noted higher *LARP1* levels in ATC cells compared to normal human thyroid cells, proposing its use as an ATC biomarker. The role of *LARP1* in cancer metastasis is also crucial, as Jiang et al.^27^ linked its irregular expression with the onset and spread of intrahepatic cholangiocarcinoma. miR‐185‐5p/miR‐214‐3p are known to inhibit tumor growth and metastasis in clear cell renal cell carcinoma by targeting lncRNA ASB16‐AS1 and suppressing *LARP1* expression.^28^ The role of *LARP1* in ATC metastasis is less defined. Our study knocked down *LARP1* in ATC to explore its effects on cell biology, revealing that *LARP1* reduction inhibited the malignant behaviors of ATC cells, EMT, and the expression of metastasis‐related proteins. Our elucidations affirmed the agency of *LARP1* in the advancement of ATC metastasis and accentuated its integral participation in the mTOR signaling pathway. The *LARP1*/mTOR axis has been shown to facilitate the proliferation and metastatic spread of epithelial and colorectal cancers.^12^, ^21^ Phosphorylated *LARP1* scaffolds mTORC1 on the 3′UTR of ribosomal protein (RP) mRNA encoding actively translating ribosomes to promote mTORC1‐dependent translation initiation induction.^29^ In this study, we found that knocking down *LARP1* significantly reduced the protein expression of mTOR and p‐mTOR. However, the decrease in p‐mTOR protein expression was not as significant as the overall mTOR protein expression. The decrease in total mTOR protein expression may indicate a general inhibition of the mTOR signaling pathway by sh‐*LARP1*, suggesting that *LARP1* can exert a pro‐tumorigenic effect through the mTOR signaling pathway. Collectively, these findings implied that the therapeutic targeting of *LARP1* or the mTOR pathway could be a promising approach to counteract the malignant metastasis in ATC.

The previous literature has reported that the extract from the TCM Chan Su, known as TBG, can impede metastasis, invasion, and EM in BC and block the mTOR signaling pathway.^30^ TBG significantly inhibited the protein expression of PLK1 in HNSCC cells, thereby suppressing the expression of G2/M phase‐associated proteins including CDK1, CDC25c, and Cyclin B1, while the inhibited PLK1 was unable to promote nuclear ectasia of CTCF, thus inhibiting the metastasis of HNSCC cells. This study demonstrated that TBG could be an effective drug for the clinical treatment of HNSCC patients.^18^ Intriguingly, *LARP1*, a keystone in the mTOR pathway's influence on cancer metastasis,^21^ caught our attention as a potential interactor with TBG. To explore this, we conducted molecular docking simulations and cellular thermal shift assays, both of which confirmed a physical binding relationship between the two. In the absence of TBG, the thermal stability of *LARP1* decreases, indicating a direct binding interaction between TBG and *LARP1*. This phenomenon may be attributed to the denaturation and inactivation of nonspecifically bound *LARP1* at higher temperatures, while the tight binding of *LARP1* with TBG enhances the stability of the *LARP1* structure, thus maintaining its activity at elevated temperatures. The interaction between TBG and *LARP1* may play a crucial role in regulating cellular signaling, proliferation, or metabolic processes. This mechanism could be important in understanding the role of *LARP1* in cellular functions. Subsequently, we investigated the effects of TBG and *LARP1* on ATC and found that TBG downregulated the expression of *LARP1* in ATC cells and effectively inhibited the metastasis of ATC cells, while the overexpression of *LARP1* attenuated the inhibitory effect of TBG on ATC metastasis. The capacity of TBG to inhibit tumor metastasis has been validated in osteosarcoma^31^ and non‐small cell lung cancer,^32^ among other solid tumors. Its potent anti‐metastatic properties suggest it could be highly beneficial in ATC patient treatment, marking it as a promising therapeutic option for ATC.

Through our research, we have illuminated that TBG targets *LARP1* to suppress mTOR signaling, preventing the metastasis of ATC. These revelations provide theoretical support for elucidating the etiology of ATC metastasis and for identifying targets for diagnosis and treatment. They also herald a promising therapeutic strategy utilizing TBG, an active component from TCM, for ATC intervention. However, this study has limitations, including the need for *in vivo* studies to verify TBG's interaction with *LARP1*, the molecular mechanisms through which TBG reduces the expression of *LARP1*, and how *LARP1* regulates the expression of mTOR. The role of other mTOR pathway effectors like Akt in ATC metastasis is also an area for future studies.

## CONFLICT OF INTEREST STATEMENT

The authors declare no conflict of interest.

## Supporting information


**Figure S1.** Overexpression of *LARP1* promotes ATC cell metastasis via the mTOR pathway. (A, B) Construction of oe‐NC, oe‐*LARP1*, and oe‐*LARP1* + Rapamycin 8505C cell groups, with transfection efficiency assessed by qRT‐PCR and WB. (C) CCK‐8 assay of cell proliferation ability of oe‐NC, oe‐*LARP1*, and oe‐*LARP1* + Rapamycin 8505C cell groups. (D) Colony formation assay for the proliferation of 8505C cells. (E, F) Scratch healing and Transwell assays for the evaluation of 8505C cell migration and invasion, respectively, using crystal violet for staining. (G) Cell adhesion assay for adhesion capability, using hematoxylin for staining. (H) WB analysis of EMT‐related proteins (E‐cadherin, Vimentin, and N‐cadherin) and metastasis‐related proteins (MMP‐2 and MMP‐9) in 8505C cells. (I) WB detection of mTOR and p‐mTOR protein expression in 8505C cells. *n* = 3 independent replicate experiments, *p < 0.05.

## Data Availability

The data and materials in the current study are available from the corresponding author on reasonable request.
