# Supplementary figures and images for: Telocinobufagin suppresses malignant metastasis of undifferentiated thyroid carcinoma via modulation of the LARP1 ‐mTOR pathway
Source: Kaohsiung J Med Sci. 2025 Jan 9;41(3):e12934. doi: 10.1002/kjm2.12934 (PMC11924809; doi:10.1002/kjm2.12934)

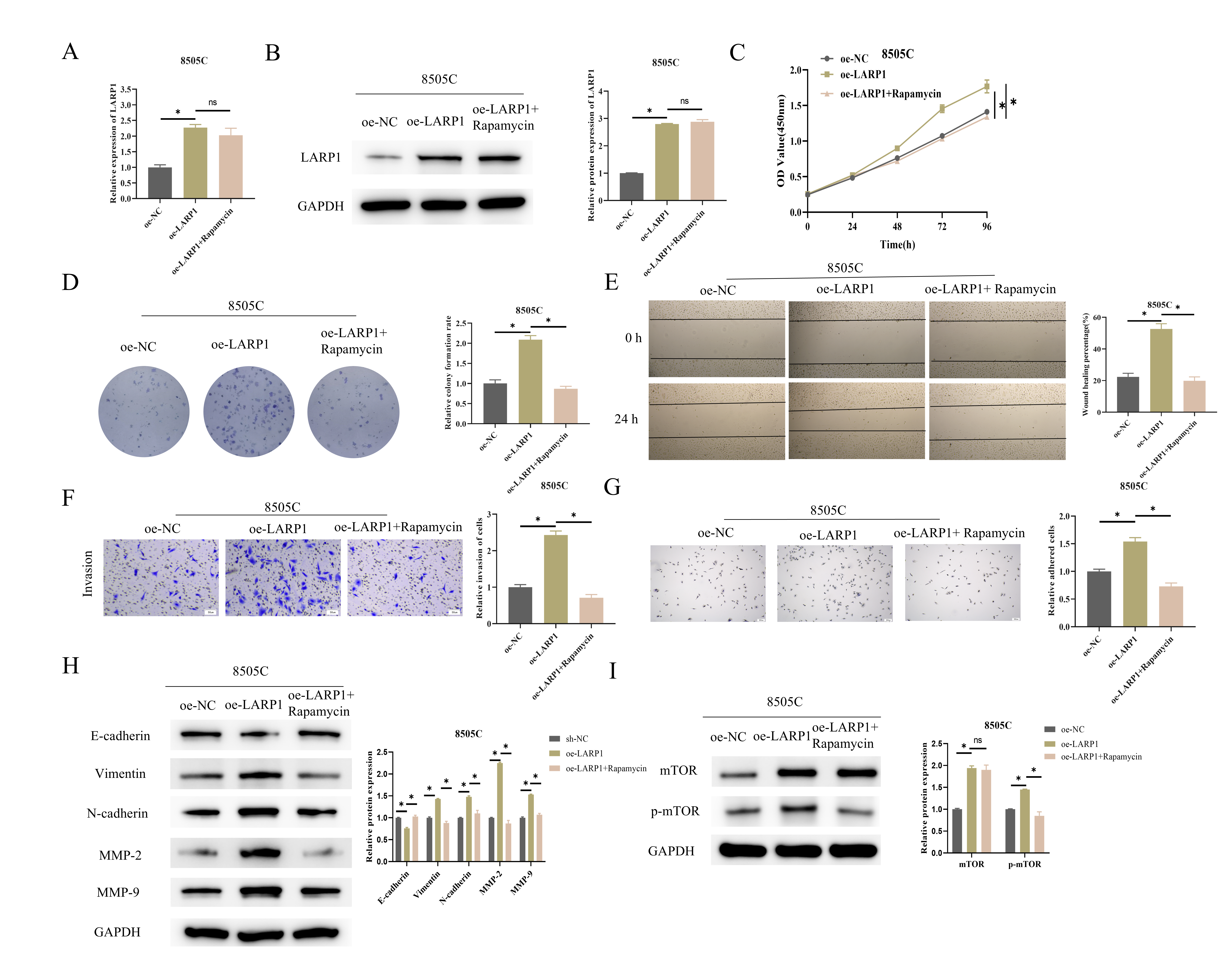

Supplement: Supplementary file 1 — Figure S1. Overexpression of LARP1 promotes ATC cell metastasis via the mTOR pathway. (A, B) Construction of oe‐NC, oe‐LARP1, and oe‐LARP1 + Rapamycin 8505C cell groups, with transfection efficiency assessed by qRT‐PCR and WB. (C) CCK‐8 assay of cell proliferation ability of oe‐NC, oe‐LARP1, and oe‐LARP1 + Rapamycin 8505C cell groups. (D) Colony formation assay for the proliferation of 8505C cells. (E, F) Scratch healing and Transwell assays for the evaluation of 8505C cell migration and invasion, respectively, using crystal violet for staining. (G) Cell adhesion assay for adhesion capability, using hematoxylin for staining. (H) WB analysis of EMT‐related proteins (E‐cadherin, Vimentin, and N‐cadherin) and metastasis‐related proteins (MMP‐2 and MMP‐9) in 8505C cells. (I) WB detection of mTOR and p‐mTOR protein expression in 8505C cells. n = 3 independent replicate experiments, *p < 0.05. [file KJM2-41-e12934-s001.tif]
